# Collection, Establishment and Assessment of Complex Human Osteocartilaginous Explants for Modeling Osteoarthritis

**DOI:** 10.3390/biomedicines12102406

**Published:** 2024-10-21

**Authors:** Camelia-Mihaela Danceanu-Zara, Adriana Petrovici, Luminita Labusca, Anca Emanuela Minuti, Cristina Stavila, Petru Plamadeala, Crina Elena Tiron, Dragoş Aniţă, Adriana Aniţă, Nicoleta Lupu

**Affiliations:** 1 National Institute of Research and Development in Technical Physics, 700050 Iasi, Romania; cdanceanu@phys-iasi.ro (C.-M.D.-Z.); aminuti@phys-iasi.ro (A.E.M.); cstavila@phys-iasi.ro (C.S.); nicole@phys-iasi.ro (N.L.); 2 Transcend Center Regional Oncology Institute, 700483 Iasi, Romania; transcendctiron@iroiasi.ro; 3 Regional Center of Advanced Research for Emerging Diseases, Zoonoses and Food Safety, Faculty of Veterinary Medicine, Iași University of Life Sciences (IULS), 8 Mihail Sadoveanu Alley, 700489 Iasi, Romania; p.adriana6@yahoo.com (A.P.); dragos_anita@yahoo.com (D.A.); adriana.anita@iuls.ro (A.A.); 4 Orthopedics and Trauma Clinic, County Emergency Hospital, 700111 Iasi, Romania; 5 Pathology Department, Saint Mary‘s Children Hospital, 700309 Iasi, Romania; p.petru@yahoo.com

**Keywords:** osteoarthritis, synovial tissue, osteocartilaginous explants, ex vivo models, sample collection

## Abstract

With the increasing burden of osteoarthritis worldwide, cost efficient and reliable models are needed to enable the development of innovative therapies or therapeutic interventions. Ex vivo models have been identified as valuable modalities in translational research, bridging the gap between in vitro and in vivo models. Osteocartilaginous explants from Osteoarthritis (OA) patients offer an exquisite opportunity for studying OA progression and testing novel therapies. We describe the protocol for establishing human osteocartilaginous explants with or without co-culture of homologous synovial tissue. Furthermore, a detailed protocol for the assessment of explanted tissue in terms of protein content using Western blot and immunohistochemistry is provided. Commentaries regarding the technique of choice, possible variations and expected results are inserted.

## 1. Introduction

Osteoarthritis (OA) is a degenerative disease that involves all joint compartments leading to the destruction of articular surfaces with consistent pain and progressive loss of function as the result [1]. The burden of OA is increasing globally, impacting individual, familial, and community-level quality of life [2]. OA treatments are limited to symptom relief, non-steroidal anti-inflammatory drugs (NSAIDs), and corticosteroids as the major components of the recommended interventions [3]. Limitations for the development of innovative therapeutics are mainly related to the complexity of the underlying pathogenic mechanisms as well as the unmet need for relevant disease models that could reflect disease phenotypes as well [4]. Models that closely reproduce complex intracellular and cell extracellular matrix interactions within all joint compartments and that can model complex tissue architecture and reflect patient stratification are necessary for testing such therapies in preclinical stages. Osteochondral explants have been described as useful modalities to study joint development and disease and to screen novel therapeutics [5]. Explanted tissues were shown to retain basic physiological functions and to react to various applied biochemical and mechanical stimuli [6,7]. The few current reports regarding explant establishment and processing are sometimes challenging to follow, occasionally failing to provide reproducible means for obtaining stable long-term culture [8,9]. Here, we describe our experience in performing long-term culture of human osteochondral explants followed by protocols dedicated to the assessment of tissular protein content using Western blot and immunohistochemistry. Protocols tailored to process mixed human bone and cartilage explanted tissues and co-culture with joint homologous synovial explants as well as expected results, alternative protocols, and comments are inserted below, grouped into three main parts devoted to the establishment and maintenance of tissue culture, protein analysis using Western blot (WB) and immunohistochemistry (IHC).

Explanted chondral tissue was used starting from the late 1990s as a modality to study cartilage metabolic changes during dynamic compression [10] or the effects of imbalanced extracellular matrix proteins on cartilage growth during development [11]. Compared to in vivo studies, explanted tissue has been recognized as a modality to provide a defined and controlled environment to study cartilage function and mechanical regulation [12]. In the last few decades, scientific as well as medical communities witnessed increasing awareness of the importance of the complexity of articular joints that need to be approached as a whole organ. The importance of investigating inter-tissue crosstalk for deciphering the mechanisms of metabolic maintenance and turnover has prompted the use of combined osteochondral grafts as models for studying articular joint metabolism, development [13], repair [14] and functioning upon different aggressors [15]. Human osteochondral explants harvested from elderly donors were found to respond to different OA-related triggers (inflammation, hypertrophy, and mechanical stress) and were proposed as reliable models for studying future therapeutic interventions [8]. Adult horse osteochondral explants with non-homologous synovial tissue were demonstrated to respond to pro-inflammatory stimuli (TNF-α, IL-1β) as well as to mechanical damage with de novo-initiated inflammation as assessed by immunohistochemistry, qPCR, and ELISA of explanted tissue and culture media, respectively [16,17]. The inclusion of synovial tissue within in vitro/ex vivo models for OA has been recognized as important in mimicking the joint inflammatory milieu modulation across tissue homeostasis, disease, and the assessment of the therapeutic response [18]. Being the fastest to respond to both local, regional as well as systemic metabolic fluctuation or injury, ex vivo models that add joint homologous synovium are only beginning to be established with few reports referring to human tissues. Osteochondral explants, especially when co-cultured with joint homologous synovium, have the advantage of offering biomimetic models that reflect both species and individual variety. Statistical analysis of data extracted from cartilage–synovium explants of multiple donors was presented as a modality to tackle donor variability and to enable multiple readouts necessary for the evaluation of therapies [19].

## 2. Protocol

### 2.1. Method 1

#### Establishment of Long-Term Culture of Human Osteochondral and Synovial Samples

M.1.1: The steps necessary for establishing human osteochondral explant culture are described below. Please note that this protocol includes co-culture with joint homologous synovial tissue; however, this step is not mandatory for performing the basic protocol. Synovial tissue might or might not be available on sample collection depending on donor particularities and surgical procedures chosen by the operating surgeon.

Samples should be collected within 2–8 h after surgery. Delayed collection up to a maximum of 24 h requires an additional step for keeping the samples in sterile condition in a refrigerator (4 °C). Keep in mind that cell viability might be affected by delayed collection of samples.

Transport of samples from the operation room to the lab requires sterile manipulation as well as sterile containers prefilled with an adequate volume of sterile PBS enough to cover the samples upon immersion. Containers should have a lid that prevents leakage of the fluid. If fluid leakage from the container is observed the sample should be considered to be contaminated and if, possible discarded. It is highly recommended to prepare washing solution as well as explant culture media beforehand since tissue manipulation is time-sensitive.

### 2.2. Materials

Cell and tissue culture media

DMEM (HG), D5671—500 mL, Sigma, Life Science, St. Louis, MO, USA

DMEM (LG), D6046—500 mL, Sigma, Life Science, St. Louis, MO, USA

L glutamine, G-7513—100 mL, Sigma, Life Science, St. Louis, MO, USA

Dexamethasone, D1881—100 mL, Sigma, Life Science, St. Louis, MO, USA

Ascorbic acid 2-Phosphate, 49752—10 G, Sigma, Life Science, St. Louis, MO, USA

L-Proline, P0380-100G, Sigma, Life Science, St. Louis, MO, USA

ITS+ supplement, I2521—5 mL, Sigma, Life Science, St. Louis, MO, USA

Sodium pyruvate, P5280—25 G, Sigma, Life Science, St. Louis, MO, USA

PBS, D8537—500 ML, Sigma, Life Science, St. Louis, MO, USA

Antibiotic 100×, A5955—20 mL, Sigma, Life Science, St. Louis, MO, USA

Serological pipettes, 5 mL, MPIP-J05-200, Thermo Fisher, Rockford, IL, USA

Serological pipettes, 10 mL, MPIP-J10-200, Thermo Fisher, Rockford, IL, USA 

Pipette tips, TIAF-1KO-096, ASTIK’S

Sterile petri dish 36.5 mm, 153066, Thermo Fisher, Kandel, Germany

Prepare stock solutions for chondrogenic media in advance. Stock solutions should be kept in the freezer, −20 °C adequate recipients tailored to the number of fresh media needed. 

Dexamethasone: 1 mm stock

Ascorbic acid 2-Phosphate: 5 mg/mL

L-Proline: 4 mg/mL

### 2.3. Equipment

Safety cabinet

Tissue culture microscope (EVOS fluorescent inverted microscope Life technologies or equivalent)

Incubator (Nuve, EC160, Ankara, Turkey)

175-cm^2^ (T-175) cell culture flasks (Thermo Fisher, Rockford, IL, USA)

Serological pipettes, 5 mL, 10 mL, 25 mL; MPIP-J05-200, MPIP-J10-200, MPIP-J25-150 Thermo Fisher, Rockford, IL, USA 

Pipette tips, TIAF-1KO-096, ASTIK’S

Polypropylene sample container sterile, 200 mL (Novolab, Geraardsbergen, Belgium or equivalent)

Sterile surgical instruments for cutting and handling (forceps, scissors) or punch biopsy (see alternative protocol below)

Sterile surface for tissue placement (can be a sterile instrument box or larger sterile Petri dish)

### 2.4. Protocol Steps

To be prepared in advance

1. Washing solution PBS with 2% antibiotics—500 mL.
2. Chondrogenic media—500 mL (Table 1).

Mix the required excipients inside the biosafety cabinet. Refrigerate at 4 °C and warm in a water bath.

1. Transport the tissues from the operating room to the lab in a thermal insulating bag.
2. Disinfect the surface of the container with 80% alcohol and place it inside the biosafety cabinet.
3. Remove the PBS used for transportation using an aspirator or a sterile serological pipette.
4. Wash tissue 3 times using PBS with 2% antibiotics at room temperature prepared in advance.
5. Place tissue on a sterile surface for macroscopic evaluation using sterile forceps. Take pictures for documenting the samples and for gross assessment of the stage of osteoarthritis (optional, if needed for the experiment).
6. Carefully cut the bone and cartilage block tissue in the needed number of specimens perpendicular to the surgical cut.
7. Place the tissue fragments within a sterile Petri dish.
8. Add chondrogenic media until fully covering the tissue.
9. For co-culture with synovial tissue, place fragmented synovial tissue inside the Petri dish.
10. Place in the incubator.

## 3. Western Blot Assessment of Osteochondral and Synovial Tissues from Human Osteochondral Explants

In the following, we introduce the protocol for performing Western blot analysis of human bone cartilage and synovial tissues from long-term cultured explants. The described protocol is performed on frozen tissues stored in liquid nitrogen after the termination of the experiment. Alternatively, fresh tissue can be used as specified below. The protocol involves the use of liquid nitrogen which should be readily available when the step involving protein extraction from tissue is planned. Please follow the safety regulations required when handling liquid nitrogen: use eye protection goggles and special gloves for handling LN recipients. Liquid nitrogen-based tissue processing for protein extraction should be performed within a fume hood. The protocol has several distinct steps listed below. The dispositives and reagents that are required for Western blot analysis are listed in Table 2 and Table 3.

### 3.1. Tissue Sample Processing

Use fresh or frozen tissue. Immerse the tissue in liquid nitrogen and plaster it. Repeat the process until you achieve a fine powder. As an alternative, automated tissue crushers and homogenizers can be used when processing tissue samples.

Add RIPA lysis buffer (5 mL/1 g tissue powder) with protease inhibitor and incubate on ice for 30 min (during incubation, swirl for five minutes three times).

Transfer to ultracentrifuge tubes and centrifuge at 14,000 rpm for 20 min at 4 °C.

Collect the supernatant.

### 3.2. Sample and BUFFERS Preparation

Sample preparation: Using a 4× sample buffer, prepare the lysate sample in microfuge tubes. Add 1× of 355 mM of 2-mercaptoethanol to the 4× sample buffer to ensure the samples are adequately reduced and denatured. The sample should then be boiled for 5 min at 95 °C under vortex. Before loading the sample into each lane, let it cool on ice for a few minutes.

To be prepared in advance:

1. Running Buffer 10×—1000 mL

144 g Glycine

30.4 g Tris Base

10 g SDS

Store up at 4 °C until use. Bring volume to 1000 mL with deionized water.

2. Running Buffer 1—1000 mL
3. Transfer Buffer—1000 mL

200 mL Tris/Glycine Buffer 10

400 mL Methanol

Bring volume to 2000 mL with deionized water. Store up at 4 °C until use.

4. Blocking Buffer

20 mL EveryBlot Blocking Buffer

Store up at 4 °C until use.

5. Washing Buffer—2000 mL

100 mL TBS 10×

1 mL Tween 20

Bring volume to 1000 mL with deionized water. Store up to 4 °C (Max 6 months).

### 3.3. Protein Quantification

Determine the protein concentration using Quick Start Bradford Protein Assay kit 1, containing 1× Dye Reagent and BSA Standard 2 mg/mL. Alternatively, protein samples can be quantified by UV absorbance as well as the BCA assay.

Pipet each standard and sample solution (volume of standard and sample: 5 µL) into separate microplate wells. 

Add 1× dye reagent (250 µL) to each well and vortex.

Incubate at room temperature for at least 5 min but no longer than 1 h at room temperature. 

Read the absorbance at 595 nm with a spectrophotometer.

### 3.4. Gel Electrophoresis

A pre-cast gel can be used (Mini-PROTEAN TGX Gels, Bio-Rad, Richmond, CA, USA) or a percentage SDS gel (Table 4 and Table 5) can be prepared based on the molecular weight of the protein of interest, using a Mini-PROTEAN System Casting Stand.

### 3.5. Protein Transfer

Use either Polyvinylidene difluoride (PVDF) or nitrocellulose membranes for the transfer of proteins.

Activate the PVDF membrane by immersing it in ethanol for 5 min.

It is recommended to leave the gel in the transfer buffer for 5–10 min after electrophoresis to equilibrate it.

Using the manufacturer’s recommendations, assemble a transfer sandwich system sequentially.

Remove all air bubbles between the gel and the membrane with a roller.

Set the voltage at 100 V for 60–90 min.

### 3.6. Membrane Blocking

Use EveryBlot Blocking buffer for 5 min, or bovine serum albumin (BSA) 5% for 1 h.

Wash the membrane five times for 5 min. with TBS-T before incubation with the primary antibody.

### 3.7. Antibody Incubation

Dilute the primary antibody in EveryBlot Blocking Solution and incubate the membrane at room temperature for 1 h with agitation. A BSA 5% (*v*/*v*) solution can also be used to dilute the primary antibody by incubating the membrane overnight at 4 °C.

Wash the membrane five times for 5 min with TBS-T to remove as much of the nonspecific bindings as possible.

Incubate membrane at room temperature with HRP-conjugated secondary antibody at the manufacturer’s recommended dilution for one hour at RT. Dilute secondary antibody in EveryBlot Blocking Solution or BSA 5%.

Wash the membrane five times for 5 min using TBS-T.

### 3.8. WB Detection and Visualization

For detection use Clarity Western ECL Blotting Substrate (Bio-Rad) for digital or film-based imaging of Western blots. The substrate mixture should be prepared in a ratio of 1:1.

Add the mixed detection solution to the membrane (7 mL for mini (7 × 8.5 cm) or 12 mL for mid-sized membrane (8.5 × 13.5 cm) and incubate for 5 min. Remove excess. 

For visualization use a digital imager (IBright FL1500 Imaging System by Thermo Fisher Scientific (Life Technologies Holdings Pte Ltd., Singapore)) or exposure to X-ray film.

### 3.9. Difficulties

WB is generally a straightforward technique; however, its execution can pose challenges, which can lead to unexpected results. The most common issues generally encountered while performing WB are lack of bands, spotty background, high background, and curved bands (Table 6). In the special situation of osteochondral tissue samples, protein extraction can be challenging due to the heterogeneous nature of the tissue (combined bone and cartilage). We found freeze drying in liquid nitrogen and manual crushing to be a good method enabling direct observation of the degree of tissue mincing; however, open manipulation of liquid nitrogen needs to be performed with caution, wearing protective goggles and gloves to avoid possible accidents.

Here, WB was performed to detect the presence of MMP-1, MMP-13, Collagen II, Perlecan, and beta-galactosidase in human osteochondral explants. Protein samples were extracted from previously liquid nitrogen-stored tissues. Protein samples (40 μg/well) and Precision Plus Protein Kaleidoscope ladder (Cat. #1610375, Bio-Rad, Richmond, CA, USA) were separated by SDS-PAGE on Mini-PROTEAN TGX Precast Gels with a 4–20% gradient (Cat. #4561093, Bio-Rad Laboratories, Richmond, CA, USA) for 60 min at 100 V in the running buffer. The frozen tissue was immersed in liquid nitrogen and plastered until it became powder and lysed using RIPA buffer (Cat. #20-188, EMD Millipore Corporation, Temecula, CA, USA) with mixed protease inhibitor solution. The total protein concentration was determined by Quick Start Bradford Protein Assay Kit 1 (5000201, Bio-Rad Laboratories, Richmond, CA, USA). Proteins were transferred to Immun-Blot PVDF membranes (#1620177, Bio-Rad Laboratories, Richmond, CA, USA) using the Mini Trans-Blot Cell for 75 min. PVDF membranes were blocked with EveryBlot Blocking buffer (Cat. #12010020, Bio-Rad, Richmond, CA, USA) for 5 min followed by incubation with specific primary antibodies (Table 7) in EveryBlot Blocking buffer for 1 h at room temperature or overnight at 4 °C. For the removal of any excess primary antibody, the membranes were washed in TBS-T three times for five minutes each, under agitation.

The secondary antibody (peroxidase-conjugated anti-rabbit IgG secondary antibody, (Sigma-Aldrich, St. Louis, MO, USA), diluted 1:500 was incubated with the membrane in EveryBlot Blocking buffer for one hour at room temperature. Next, the membranes were washed three times, for five minutes each, in TBS-T. PVDF membranes were exposed to Clarity Western ECL Substrate (Bio-Rad) for 5 min at room temperature in the dark and visualized using an IBright FL1500 Imaging System by Thermo Fisher Scientific (Life Technologies Holdings Pte Ltd., Singapore).

## 4. Immunohistochemistry in Human Osteocartilaginous and Synovial Explant Tissue

This method describes the protocol for performing immunohistochemistry (IHC) analysis of human osteocartilaginous and synovial tissues from long-term cultured explants. IHC is a technique used to visualize the presence and localization of specific proteins in tissues. The protocol is performed on fixed tissue. Alternatively, fresh tissue can be used.

The IHC reactions were realized using a hybrid protocol, as follows: deparaffinization and rehydration were performed on Leica Autostainer XL (Leica Biosystems Nussloch GmbH, Nussloch, Germany); epitope retrieval, blocking, and primary antibody incubation were performed manually, in the hood; detection and counterstain on Ventana GX Benchmark (Ventana Medical Systems, Inc., Tucson, AZ, USA); dehydration and clearing on Leica Autostainer XL; mounting manually.

Note! We created this hybrid protocol for tissue integrity preservation given that our trials to perform whole IHC on Ventana GX Benchmark failed (the slides had no more tissue at the end of the staining cycle, probably because of the deparaffinization and washing steps). Replacing some steps from the benchmark’s protocol with manual steps was our solution for this problem.

The Ventana GX Benchmark is an automated IHC slide staining system that improves the quality, consistency, and reproducibility of stains and can lead to easier-to-read slides with reduced variability and fewer manual steps. This can result in a faster turnaround time and true results with higher throughput. Below is a general protocol for performing IHC on human osteocartilaginous and synovial explant tissue.

IMPORTANT! For IHC, paraffin-embedded osteocartilaginous tissue samples from normal matched joints (non-osteoarthritic) donors should be available at the beginning of the protocol as a negative control. Also, a positive tissue control must be run with every staining procedure performed. This tissue may contain both positive and negative staining cells or tissue components and serve as both the positive and negative control tissue. Control tissues should be fresh autopsy, biopsy, or surgical specimens prepared or fixed as soon as possible in a manner identical to the test sections.

### 4.1. Materials, Reagents, and Solutions

This section outlines the necessary equipment, reagents, and solutions required for performing immunohistochemistry (IHC). Each of the items should be prepared in advance, and the manufacturers and locations are provided for clarity.

The following equipment is essential for performing IHC:

- Tissue Processor: HistoCore Pearl (Leica Biosystems, Nussloch, Germany)
- Paraffin Embedding Station: HistoCore Arcadia C + H (Leica Biosystems, Nussloch, Germany)
- Microtome: HistoCore AS (Leica Biosystems, Nussloch, Germany)
- Autostainer: Autostainer XL (Leica Biosystems, Nussloch, Germany)
- Automated IHC Benchmark: Ventana GX Benchmark (Roche Diagnostics, Risch, Switzerland)
- Slide Scanner: EasyScan Pro 6 (Motic, Xiamen, China)
- Filtration Fume Hood: Cruma G-5 (Cruma, Barcelona, Spain)

Miscellaneous Equipment:

- Slides moisture chamber
- Pipettes
- Embedding cassettes
- Staining jars and racks
- Superfrost Adhesion Microscope Slides (TOMO^®^, Cat. No. 11 10748-166)
- Cover glass (sufficient to cover tissue sections, such as VWR, Cat. No. 48393-060)
- Barcode labels (Roche, Cat. No. 05247829001)
- Prep Kits for BenchMark Instruments (Roche, Cat. Nos. 05275814001, 05275822001, 05275849001, 05275857001, 05275865001)
- IHC Prep Kits for Primary Antibody Dispenser (Roche, Cat. No. 1637700)

#### Reagents and Solutions for IHC

The following reagents and solutions are required for IHC preparation:

- Neutral Buffered Formalin (10% *v*/*v*): Sigma (Sigma-Aldrich, USA, Cat. No. HT501128)
- EDTA (0.07%): Sigma-Aldrich, Cat. No. E4884, CAS N° 6381-92-6
- Ethanol: Available in different concentrations:
  ✓ 70% (*v*/*v*)
  ✓ 80% (*v*/*v*)
  ✓ 95% (*v*/*v*)
  ✓ 99.8% (*v*/*v*)

- Xylene or substituent
- Paraffin (55–60%)
- Citrate Buffer (pH 6.0)
- Phosphate-buffered saline (PBS)
- Bovine Serum Albumin (BSA)
- EZ Prep Concentrate (10×): Roche, Cat. No. 950-102/05279771001
- Reaction Buffer Concentrate (10×): Roche, Cat. No. 950-300/05353955001
- LCS (Predilute): Roche, Cat. No. 650-010/05264839001 for BenchMark XT and GX instruments
- Cell Conditioning Solution (CC1): Roche, Cat. No. 950-124/05279801001 for BenchMark XT and GX instruments
- Hematoxylin II Counterstain: Roche, Cat. No. 790-2208/05277965001
- Bluing Reagent: Roche, Cat. No. 760-2037/05266769001
- Permanent Mounting Medium: Permount™ (Fisher Scientific, USA, Cat. No. SP15-500 or equivalent)
- Primary Antibodies:
- MMP13 Recombinant Polyclonal Antibody (3HCLC), Thermo Fisher Scientific, Cat. No. 710311
- Perlecan Monoclonal Antibody (A7L6), Thermo Fisher Scientific, Cat. No. MA1-06821
- Collagen II Polyclonal Antibody, Thermo Fisher Scientific, Cat. No. PA1-26206
- CD68 Antibody, Thermo Fisher Scientific, Cat. No. MA5-13324
- Beta Galactosidase Polyclonal Antibody, Thermo Fisher Scientific, Cat. No. PA5-102503
- ultraView Universal DAB Detection Kit: Ventana, Cat. No. 760-500
- Wash buffer
- Slide mounting media

### 4.2. Tissue Preparation for IHC of Osteochondral and Synovial Tissue

Obtain fresh (non-frozen, non-paraffin embedded) osteocartilaginous and synovial explant tissue at the end of the explant experiment. Fix the tissue immediately in 10% neutral buffered formalin solution for 24 h in fit-sized recipients.

For further processing, osteochondral tissues must be submitted to the decalcification process. Osteochondral tissue is removed from the fixation solution and washed ×5 with ddH_2_O. An adequate amount of 0.5 M EDTA, pH 8, to cover each sample must be placed in 50 mL polypropylene tubes and left on the bench at room temperature for 2–3 days. Every 2–3 days, the solution must be replaced with a fresh one. Samples must be checked two times a day using folding and a stilet to manually probe the softening produced by decalcification. Depending on the thickness of the tissue, an average of 5–7 days is needed for complete decalcification. This step is only required for bone-containing tissues.

Process the tissue using a tissue processor to dehydrate, clear, and infiltrate with paraffin. Synovial tissue and decalcified osteochondral tissues were dehydrated stepwise: 70% ethanol, two changes—1 h each; 80% ethanol, two changes—1 h each; 95% ethanol, two changes—1 h each; 99.8% ethanol, three changes—1 h each; xylene, three changes—1 h each; paraffin wax (56–58 °C), three changes—1.5 h each.

Embed the tissue in paraffin blocks as follows: approximately a quarter of a large base mold must be filled with paraffin wax and the sample positioned as required with tweezers. While holding the sample in place, the mold must be moved to a cold plate until the wax solidifies enough to hold the sample naturally. The mold is then filled with wax until it covers the entire sample. Finally, the block is left on the cold plate for 30–60 min until it is solid enough to be removed from the metal or plastic mold. The sample can be removed by banging the metal casing on the bench a couple of times to loosen the paraffin-embedded sample.

Section the tissue at 4–5 µm thickness using a microtome and mount on positively charged slides (SuperFrost Plus Microscope Slides TermoFisher).

### 4.3. Slide Preparation and Primary Antibody Incubation

Dry the slides in an oven at 53–65 °C for at least 2 h (but no longer than 24 h).

Allow the slides to cool to room temperature.

Deparaffinize the tissue sections using xylene or substituent.

Rehydrate the tissue sections with graded alcohol baths (99,8% ethanol, 96% ethanol, 85% ethanol, 70% ethanol) to dH_2_O.

Note! For deparaffinization and rehydration, we used an autostainer (Autostainer XL, Leica). Also, manual procedures can be performed.

Incubate the slides in Citrate Buffer (pH 6.0) at 100° Celsius and leave for 30 min for epitope retrieval.

Wash three times in PBS for 5 min each.

Prepare a blocking buffer with 3% BSA and PBS and incubate the slides for 30 min.

Do not wash the slides after the blocking step.

Incubate the slides with the primary antibody in the slide’s moisture chamber for the required time and appropriate temperature (Table 8).

Wash the slides in PBS three times for 5 min each.

### 4.4. Automated IHC—Secondary Antibody Incubation and Counterstain on Ventana GX Benchmark

Turn on the Ventana GX Benchmark and initialize the system as per the manufacturer’s instructions.

Load the required reagents, including the detection system, and other necessary reagents into the machine (Table 9).

Place the tissue slides in the slide holder (Table 9).

Select the appropriate staining protocol on the machine interface.

Start the automated staining process.

Detection—a detection system, such as DAB (3,3′-Diaminobenzidine), will be applied to visualize the bound antibodies.

Counterstaining—the tissue sections will be counterstained with hematoxylin to visualize the nuclei. Bluing reagent is used as an enhancer.

### 4.5. Dehydration and Mounting

The sections will be dehydrated through graded alcohols to xylene or xylene substitutes using the Autostainer XL from Leica.

Apply a coverslip using a permanent mounting medium.

### 4.6. Analysis and Storage

Examine the stained slides under a light microscope and acquire the images for quantification.

Note! A digital scanning system can be used for the examination of the slides. We used a Motic Easy Scan Pro 6.

Document and analyze the staining pattern, intensity, and localization.

Store the stained slides in a slide box at room temperature, away from direct sunlight.

Note! Always refer to the antibody datasheet and the Ventana GX Benchmark user manual for specific instructions and recommendations. Optimization may be required for specific antibodies or tissue types.

Safety Precautions! Always wear appropriate personal protective equipment (PPE) when handling chemicals and biological samples. Dispose of waste materials according to local regulations.

### 4.7. Difficulties

For IHC, the fixation and decalcification process for epitope preservation is essential. The tissue must not be kept in formaldehyde or other types of fixators for too long because the proteins are destroyed. Depending on the tissue type and dimensions, a fixation of a maximum of 24 h should be enough. The usual fixating solution is 10% formaldehyde, but a very good alternative is 4% paraformaldehyde (PFA) which is more indicated for proteins and mRNA detection. The 4% PFA solution should be freshly prepared by heating it at 55–60 °C and agitating it for a few minutes. NaOH must be added for the complete dissolution of the PFA.

The decalcification step is another crucial one also for preventing the destruction of our interest proteins, not only for the tissue preparation for the cutting at the microtome. The most common protocol for removing calcium from bones is with 0.5 M EDTA, pH 8.

If the tissue does not stay on the slide until the end of the reaction, the causes are multiple. The first assay is to re-cut into more thin sections and make sure the slides we are using are adhesive and within the shelf life. The second step here is to use the proper water bath temperature when taking the section on the slide and not immerse the slide too much time or more than once.

The second assay to do is re-including the tissue. The blocks must be melted and left in paraffin at 56–58 °C for 2 h. After solidification, we must begin to cut the block until we arrive at the tissue, take the block in a glass of water (room temperature) for 5 min, and then leave the block in a freezer for 5–10 min. When cutting again, it should be 2 um.

The last assay, if you have conserved another sample of the same tissue, is to include again using the right protocol. A list of possible problems and their causes is included in Table 10.

## 5. Representative Results

M1. Explants must maintain their morphological appearance during the culture without peeling off or maceration. A slight change in color with whitening or yellowish appearance is likely to appear with time. A good practice is to regularly check the eventual presence of migrated cells within the dish under an optical microscope; serum-free media should account for the absence of cell migration and retention of cellular elements within the explant. 

If harvesting cell culture media is necessary for the experiment (for cytokine and/or growth factor detection), an appropriate amount of explant-conditioned media should be harvested and stored accordingly for further investigation protocols (ELISA, PCR).

**Figure 1:**
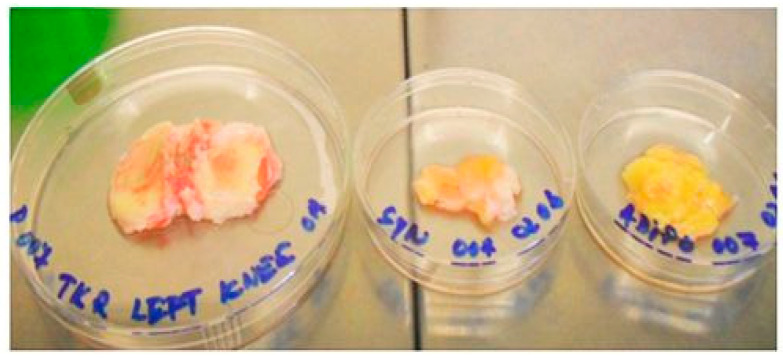
Upper tibia osteochondral block cut with synovial and adipose tissue from one donor.

Examples of surgical pieces consisting of cartilage, bone and synovial tissue obtained from total knee arthroplasty (TKA) are given below (Figure 1).

**Figure 2:**
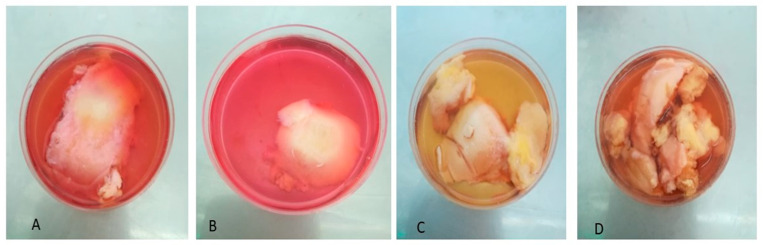
Osteochondral explants gross aspect after 30 days in explant culture: (**A**,**B**) explants without synovial tissue co-culture; (**C**,**D**) explants with synovial tissue co-culture.

We used the ICRS recommendation [20] for scoring the severity of OA lesions to match with the histological assessment of OA grading [13,21]. We found the initial marking of OA severity on the top of the bulk operative piece useful (Figure 2A–D) as it helped us to plan to harvest the explants from zones with similar degrees of OA severity and, therefore, cartilage and bone damage, for the consistency of the results. Conversely, for the situation of explanted synovial tissue, an initial gross assessment of the disease severity [22,23,24] was not performed due to tissue fragmentation and relative scarcity.

**Figure 3:**
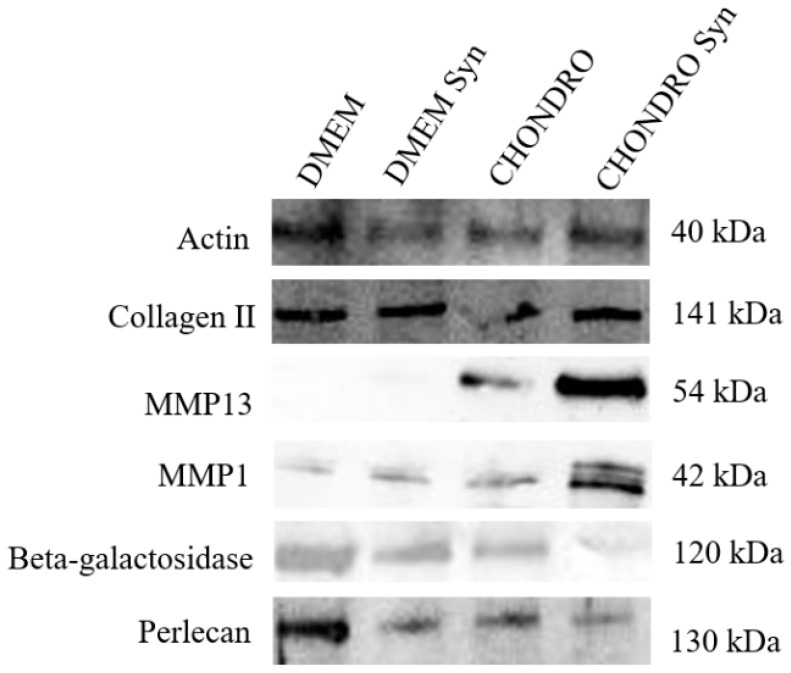
Cropped images representative of Western blot analysis showing the expression of Collagen type II, MMP-13, MMP-1, beta-galactosidase, Perlecan, and Actin from osteochondral explants cultured for 30 days with two different media: without synovial tissue co-culture and with synovial tissue co-culture.

We present the WB results (Figure 3) for detecting two matrix-associated proteins (Collagen type II and Perlecan) normally present in hyaline articular joint cartilage, two proteases involved in cartilage matrix degradation during pathologic cartilage maintenance in OA (MMP-1 and MMP-13) and one senescence-associated enzyme (beta-galactosidase) using WB, as described in M2.

## 6. Discussion and Conclusions

Compared to other preclinical models, explanted tissues are more complex than 2D or 3D culture or co-culture systems while maintaining the advantage of being kept in controlled environments of known variability. Physical and chemical parameters influencing such cultures can be strictly monitored and recorded. Explanted tissues in organotypic cultures are straightforward to maintain over extended periods within either manual or automated culture maintenance systems. At the end of the experiment, the explanted tissue is amenable to high throughput analysis using an automated workflow (see below for immunohistochemistry). Even though systemic interactions within animal models cannot be accurately reproduced, the advantage of being species-specific or even personalized offers them consistent relevance in the context of the quest for precision medicine-tailored therapies. Compared to cell culture, explants retain the characteristic architecture, microtopographic cues, and extracellular matrix components native to the respective tissue. This allows for a more accurate prediction of the response elicited by the tested therapeutic intervention. There is a constant increase in high throughput quantitative techniques for interrogating time-dependent conditioned media-released signaling molecules and/or extracellular vesicles from explant cultures [25]. Explant tissue in itself can be assessed upon termination of the experiment both structurally (cell and ECM components content) and functionally (the presence or absence of signaling molecules such as cytokines, growth factors, and tissue-specific enzymes). When intended for drug testing, an estimation of the number of replicates is advisable to ascertain tissue availability. In the case of human samples, ethical approval and donor consent should be performed preferably before starting the experiment. There are a few examples in the current published literature of experiments performed on human osteochondral tissue explants in combination with homologous synovial tissue. Since synovial tissue is a major component influencing joint turnover and metabolism, its presence is crucial for a model that attempts to mimic joint organ function as closely as possible. Being a large synovial joint, knee function and pathology are most frequently studied in ex vivo models [26]. TKA surgery offers the opportunity to collect enough osteocartilaginous tissue that preserves tissue architecture as well as other tissues that compose the joint such as the synovium and adipose tissue. The process of sample collection during TKA would require close collaboration with the surgical team to label and identify their position within the native joint and correlate with the macroscopic aspect and OA staging.

Western blotting is primarily used to detect and quantify specific proteins within a tissue sample. This is crucial for understanding the molecular composition of the tissue and for assessing changes in protein expression levels under different experimental conditions or in disease states. Western blotting allows for the specific detection of individual proteins or post-translational modifications (e.g., phosphorylation, glycosylation) within the tissue. It is often used to validate the presence or absence of a specific protein within the explant tissue, confirming the results obtained from other techniques like immunohistochemistry or PCR [27]. The name ‘Western’ blot was first coined by Burnette in 1981 after the eponymous Southern blot was already in use for DNA detection and the consequent coinage of the Northern blot in 1977 for RNA detection based on similar principles [28]. Western blot (WB), also known as immunoblot, involves separating proteins according to molecular weight and identifying them by gel electrophoresis. Using this approach, the separated proteins are transferred to a protein-binding membrane and detected with an antibody specific to the target protein. WB’s main advantages consist of sensitivity, which allows it to detect protein levels as small as 0.1 nanograms, and the specificity induced by the antigen-antibody interaction. The WB technique has several disadvantages and limitations, some of them being the complexity of the technique, which can be laborious and time-consuming; due to insufficient transfer, it can cause inaccurate bands or the absence of bands, as well as being resource intensive [29,30].

Semi-quantitative WB applications have become increasingly popular in biomedical applications over the last few decades. However, issues such as the selection of the appropriate normalization, sample preparation, determination of the calibration curve for antibodies and proteins to be tested, prevention of signal saturation, and issues with quantification of targeted protein signal intensity add to the technique’s complexity [31]. The WB technique is largely used for disease diagnosis (e.g., HIV, Lyme disease, hepatitis B) as well as in research for the detection of target proteins in biological samples (cell cultures, tissue extracts or homogenates, culture and/or conditioned media) [32]. There are few reports on using WB for protein detection in explanted osteochondral tissue samples. Tailoring protocols for protein extraction, antibody quantification, and incubation timing are often needed and seldom clarified in the existing literature (see below).

Western blots can be challenging to perform and obtain accurate results due to the multiple essential steps required. Results can be adversely affected by a momentary imbalance at any level of the process. We found that most published articles do not include the key parameters necessary for reproducing Western blot results based on common conditions. These critical parameters include (i) obtaining a qualitative lysate using a suitable homogenization method such as ultrasonication, mechanical homogenization, French press, or manual grinding, (ii) selecting high-quality primary and secondary antibodies, a substantial percentage (>50%) of commercial antibodies are of poor quality and also there is a lack of specific antibodies such that many protein targets are not able to be explored [33], (iii) optimizing the number of loaded proteins, to avoid under- or overloading protein samples, (iv) choosing the right blocking agent, and (v) optimizing antibody dilution.

Osteochondral explants were previously identified as reliable models for studying developmental and diseased articular joint stages [34]. Extracellular matrix proteins and tissue-associated catabolic enzymes involved in tissue maturation, maintenance, and degeneration are of interest to such studies. Classically, the identification of key ECM components (collagen II, GAGs) was identified using biochemical assays [35]. WB can find a place between analytical biochemical tests and proteomics techniques. Liquid chromatography–tandem mass spectrometry (LC–MS/MS) was used to analyze explant ECM composition by blasting measured peptide masses against a database of protein fragmentation patterns [36]. We propose WB could be used to validate proteomics results in novel sample sets as a standalone test or in comparison with IHC (Table 11). Herein, collagen type II, Perlecan, MMP-13, MMP-1, beta-galactosidase explant protein content, and Actin (as a loading control) were assessed (Figure 3). The osteochondral explants were cultured for 30 days in two different media, Dulbecco’s minimum essential medium (DMEM) and chondrogenic media (DMEM (high glucose-HG)) as previously mentioned (see protocol I), with and without synovial tissue co-culture. Collagen type II and Perlecan are the most significant constituents of the cartilage ECM with a critical role in cartilage biomechanics. A decrease in Col II and Perlecan content is associated with cartilage stiffness, decreased cellularity, or cartilage dysplasia [37,38]. We could detect a difference between samples kept in DMEM and Chondrogenic media in terms of ECM protein presence. The results illustrated that Perlecan displayed a noticeably increased level in explants maintained in DMEM than those maintained in chondrogenic media. Collagen II expression was similar in all conditions, apparently slightly increased in samples co-incubated with synovial tissue and less present in samples kept in DMEM. Matrix metalloproteinases MMP-1 and MMP-13 are the interstitial collagenases responsible for the degradation of type I and II collagen, with a significant role in osteoarthritis progression. In this study, MMP-1 and MMP-13 expression was more evident in samples co-cultured with synovial tissue explants, whereas weak MMP-1 expression was observed in the remaining samples. It is known that MMP-13 has a limited expression level in connective tissue when compared to other secreted collagenases [39,40,41].

Beta-galactosidase is less present in samples kept in chondrogenic media. Recently, senescence and senescence-associated phenotypes in articular chondrocytes have been associated with OA progression, and methods for decreasing cellular senescence are sought as potential OA novel therapies [42]. Using DMEM as explant culture media maintains beta-galactosidase expression while chondrogenic media might attenuate it, a fact that needs to be remembered when modeling senescence modulator therapies. Synovial tissue presence appears to be essential in mimicking the inflammatory and degradative milieu in explant models. Indeed, MMP isoform presence is more obvious in samples co-cultured with synovial tissue explants.

A crucial tool for understanding the complex architecture of osteochondral tissues is IHC. Through the use of certain antibodies directed against different cellular markers, it is possible to identify the geographical distribution of different cell types inside the explant.

**Figure 4:**
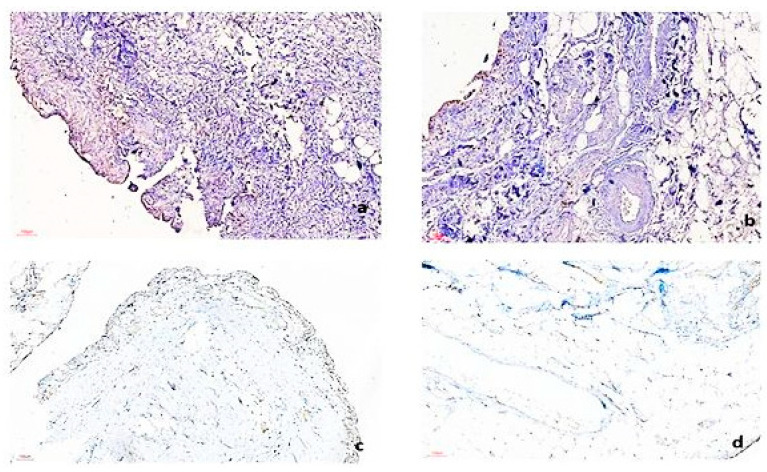
Immunohistology pictures for CD68 on explant synovial samples (brown= CD68 positive cells): (**a**) explanted synovial samples in DMEM, Scale bar = 100 µm; (**b**) explanted synovial samples in Chondrogenic media, Scale bar = 100 µm; (**c**,**d**) control synovial samples from age-matched non-OA patient Scale bar = 100 µm.

IHC is an important technique in our protocol for investigating OA within osteochondral explants. However, successful implementation of IHC in OA research requires addressing various technical challenges and troubleshooting strategies to ensure reliable and reproducible results (Table 11). To detect cellular immune reactivity in the explanted synovium, we conducted immunohistochemical (IHC) staining for CD68 (Figure 4), followed by a quantitative assessment of CD68-positive elements using semiautomated image analysis. Compared to control tissue (a synovial sample from an age-matched OA-free donor undergoing knee surgery for a traumatic event), the explants kept in CHONDRO media had a higher percentage of CD68-positive cells relative to the total image area.

**Figure 5:**
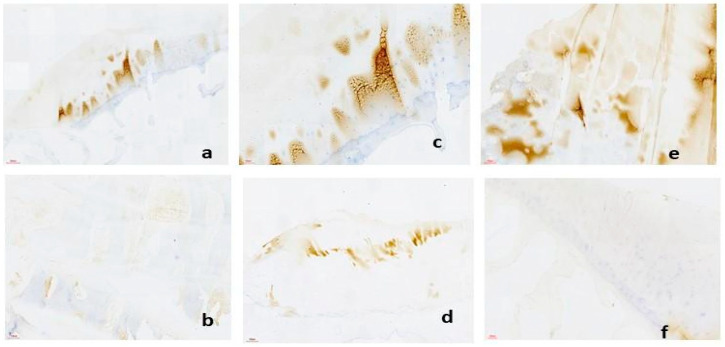
Immunohistology for Collagen II (brown= Collagen II positive): (**a**) explanted osteochondral tissues in DMEM, Scale bar = 100 µm; (**b**) explanted osteochondral tissues in DMEM plus synovium co-culture, Scale bar = 100 µm; (**c**) explanted osteochondral tissues in Chondrogenic media, Scale bar = 100 µm; (**d**) explanted osteochondral tissues in chondrogenic media plus synovial co-culture, Scale bar = 100 µm; (**e**) control samples—non-osteoarthritic osteocartilaginous tissue, Scale bar = 100 µm; (**f**) negative antibody control, Scale bar = 100 µm.

**Figure 6:**
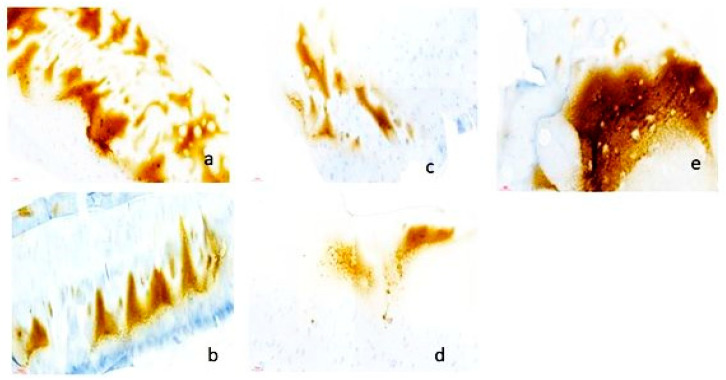
Immunohistology for Perlecan (brown= Perlecan positive): (**a**) explanted osteochondral tissues in DMEM, Scale bar = 100 µm; (**b**) explanted osteochondral tissues in DMEM plus synovium co-culture, Scale bar = 100 µm; (**c**) explanted osteochondral tissues in Chondrogenic media, Scale bar = 100 µm; (**d**) explanted osteochondral tissues in chondrogenic media plus synovial co-culture, Scale bar = 100 µm; (**e**) control samples—non-osteoarthritic osteocartilaginous tissue, Scale bar = 100 µm.

Within OA-affected osteochondral explants, cartilaginous matrices were visible thanks to the use of Collagen II (Figure 5) and Perlecan (Figure 6) as markers. Our results are consistent with other research showing changes in Collagen II distribution and integrity in OA cartilage [43,44]. We improved our understanding of OA pathophysiology by elucidating the disarray and loss of Collagen II-rich extracellular matrix that characterize OA cartilage degradation using precision staining techniques (Table 11).

Another crucial marker in our IHC investigations was Perlecan, a significant proteoglycan in the extracellular matrix that showed changes in distribution among OA-affected tissues. Our results support earlier studies that showed variations in Perlecan expression and localization in subchondral bone and OA cartilage [45,46]. We were able to learn more about the breakdown of the Perlecan-rich matrix network in OA joints by using Perlecan labeling, which advanced our knowledge of the molecular milieu controlling OA pathophysiology.

**Figure 7:**
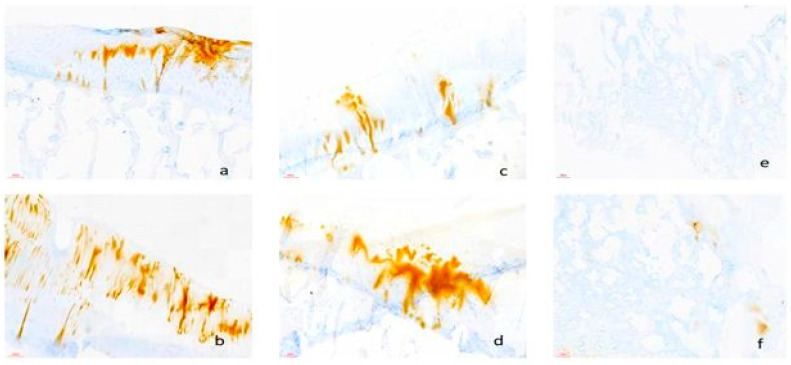
Immunohistology for MMP-13 (brown = MMP-13 positive): (**a**) explanted osteochondral tissues in DMEM, Scale bar = 100 µm; (**b**) explanted osteochondral tissues in DMEM plus synovium co-culture, Scale bar = 100 µm; (**c**) explanted osteochondral tissues in Chondrogenic media, Scale bar = 100 µm; (**d**) explanted osteochondral tissues in chondrogenic media plus synovial co-culture, Scale bar = 100 µm; (**e**) control samples—non-osteoarthritic osteocartilaginous tissue, Scale bar = 100 µm; (**f**) negative antibody control, Scale bar = 100 µm.

Additionally, measuring MMP-1 and MMP-13 (Figure 7) expression shed light on dysregulated tissue remodeling mechanisms in osteochondral explants impacted by OA. Our research showed that MMPs were overexpressed in OA joints, which is consistent with previous research [47,48]. MMPs are also linked to cartilage degradation and matrix turnover. IHC makes it easier to characterize the molecular pathways causing cartilage degradation in OA by mapping MMP expression patterns. This provides prospective targets for therapeutic therapies meant to block MMP activity.

Furthermore, the evaluation of beta-galactosidase activity using IHC revealed information about cellular senescence and aging-related alterations in osteochondral tissues affected by osteoarthritis. Our research builds on earlier studies that found a connection between tissue deterioration and senescent cell formation in OA joints and beta-galactosidase activity [49,50]. IHC made it easier to identify senescent cell populations in OA-affected tissues by identifying Beta-galactosidase-positive cells, suggesting their possible role in the development of OA and joint degeneration.

Regarding the difficulties encountered in the optimization of the protocol, it is well known that the osteocartilage from OA is difficult to work with due to the mixed bone tissue composition and the tissue heterogeneity in terms of histology processing especially in OA. Human osteochondral explants exhibit inherent variability due to donor age, joint location, and OA stages. Inadequate tissue fixation or processing can compromise antigen preservation and cause poor staining quality. Osteochondral explants are inherently heterogeneous structures with varying tissue densities and compositions, making it challenging to achieve uniform fixation and preservation of antigenicity across the entire sample. Suboptimal fixation can result in tissue distortion, protein denaturation, and loss of antigenic epitopes, leading to poor staining quality and unreliable data [43,44].

OA-affected tissues often exhibit alterations in extracellular matrix composition and tissue structure, posing challenges for antibody penetration and antigen retrieval. To address this, optimizing tissue processing protocols, including fixation, and embedding techniques, is essential to preserve tissue integrity and antigenicity. Additionally, implementing antigen retrieval methods such as heat-induced epitope retrieval can enhance antibody binding and improve staining consistency.

Achieving optimal staining conditions is essential for maximizing the signal-to-noise ratio and minimizing background staining in IHC experiments. Factors such as antibody concentration, incubation time, and detection systems should be carefully optimized to ensure robust and reproducible staining results.

Osteochondral explants derived from OA joints often exhibit heterogeneous tissue composition and cellular phenotypes, complicating IHC interpretation. To address this challenge, selecting representative tissue sections for analysis and implementing systematic sampling strategies combined with statistical interpretation was proposed as a method to minimize variability between samples. Additionally, utilizing multiple complementary markers permits comprehensive characterization of the cellular and molecular changes associated with OA pathology. We propose that building up multiplex panels of antibodies for both WB and IHC evaluation could be of use to standardize the assessment of tissue reactivity in the case of osteochondral samples employed as ex vivo models for testing novel OA therapies.

**Notes:**

- Ensure all procedures were performed under sterile conditions to maintain sample integrity.
- Documentation at each step is crucial for reproducibility and assessment accuracy.
- Adjust timings and concentrations based on specific sample conditions and experimental needs.

This protocol aims to provide a comprehensive framework for the systematic collection, maintenance, and assessment of human osteochondral samples, facilitating high-fidelity ex vivo models for studying OA and evaluating therapeutic interventions.

## Figures and Tables

**Table 1:** Reagent concentration for chondrogenic media.

Reagent	Volume (To Make 500 mL)	Final Concentration
DMEM (HG)	485 mL	500 mL
Dexamethasone 1 mM	50 µL	100 nM
Ascorbic acid 2-P: 5 mg/mL	5 mL	50 µg/mL
L-Proline: 4 mg/mL	5 mL	40 µg/mL
ITS+ supplement	5 mL	6.25 μg/mL bovine insulin 6.25 μg/mL transferrin 6.25 μg/mL selenous acid 5.33 μg/mL linoleic acid 1.25 mg/mL BSA
Sodium pyruvate	5 mL	1 mM
Antibiotic	5 mL	2%

**Table 2:** List of Western blot dispositives.

Fume Hood	
Centrifuge	
IBright FL1500 Imaging System	Invitrogen by Thermo Fisher Scientific (Life Technologies Holdings Pte Ltd., Singapore)
Magnetic stirrer	BioSanRiga, Latvia
Gel electrophoresis system	Bio-Rad, Richmond, California
Mini-PROTEAN System Casting Stand	Bio-Rad, Richmond, CA, USA
Power source	Bio-Rad, Richmond, CA, USA
Heat block	BioSan, Riga, Latvia
Balance	Kern ABJ80-4NM, Balingen, Germany
Vortexer	BioSan, Riga, Latvia

**Table 3:** List of reagents used in Western blot.

Lysis buffer (RIPA)	Bio-Rad, Richmond, CA, USA
4× Laemmli Sample Buffer	Bio-Rad, Richmond, CA, USA
2 Mercaptoethanol	Bio-Rad, Richmond, CA, USA
Mini-PROTEAN TGX Gels	Bio-Rad, Richmond, CA, USA
PVDF membranes	Bio-Rad, Richmond, CA, USA
Filter paper	Bio-Rad, Richmond, CA, USA
Sponges	Bio-Rad, Richmond, CA, USA
Washing buffer	Bio-Rad, Richmond, CA, USA
Loading buffer	Bio-Rad, Richmond, CA, USA
Transfer buffer	Bio-Rad, Richmond, CA, USA
EveryBlot Blocking buffer	Bio-Rad, Richmond, CA, USA
Methanol 100%	Bio-Rad, Richmond, CA, USA
Clarity Western ECL Substrate	Bio-Rad, Richmond, CA, USA
MMP 1 Recombinant Polyclonal Antibody	PA5-27210, Thermo Fisher Scientific, Rockford, IL, USA
MMP13 Recombinant Polyclonal Antibody	3HCLC, 710311, Thermo Fisher Scientific, Rockford, IL, USA
Collagen II Polyclonal Antibody	PA1-26206, Thermo Fisher Scientific, Rockford, IL, USA
beta Galactosidase Polyclonal Antibody	PA5-102503, Thermo Fisher Scientific, Rockford, IL, USA

**Table 4:** List of stacking gel components.

Stacking Gel Components	1 Gel
Acrylamide/Bis Solution 40%	0.5 mL
0.5 M Tris HCl buffer pH 6.8	1.25 mL
dH_2_O	3.2 mL
SDS 10%	50 µL
APS 10%	50 µL
TEMED	5 µL

**Table 5:** List of running gel components.

Running Gel Components	8%	10%	12%	15%
Acrylamide/Bis Solution 40%	2 mL	2.5 mL	3 mL	3.75 mL
1.5 M Tris HCl buffer pH 8.8	2.5 mL	2.5 mL	2.5 mL	2.5 mL
dH_2_O	5.35 mL	4.85 mL	4.35 mL	3.6 mL
SDS 10%	100 µL	100 µL	100 µL	100 µL
APS 10%	100 µL	100 µL	100 µL	100 µL
TEMED	10 µL	10 µL	10 µL	10 µL

**Table 6:** Troubleshooting guide for Western blotting analysis.

Problem	Possible Cause	Solution
Lack of bands	Over-transfer or under-transfer Sodium azide may be present in buffers	Check and optimize the conditions of transfer. Use azide-free buffers
Spotty background	Aggregated secondary antibody Contamination	Filter to remove aggregates Use fresh buffers
High background	High concentration of the antibody	Optimize concentrations Use fresh buffers Increasing the washing time
Curved bands	Gel overheated	Reduce voltage Run gel at 4 °C

**Table 7:** Table showing the basic information about primary and secondary antibodies used in the Western blot technique.

Antibody Name	Supplier	Type	Host	Dilution
MMP-1(PA5-27210)	Thermo Fisher Scientific, Rockford, IL, USA	Primary antibody/Polyclonal	Rabbit/IgG	1:1000
MMP-13 (710311)	Thermo Fisher Scientific, Rockford, IL, USA	Primary antibody/Oligoclonal	Rabbit/IgG	1:1000
Collagen II(PA1-26206)	Thermo Fisher Scientific, Rockford, IL, USA	Primary antibody/Polyclonal	Rabbit/IgG	1:1000
Beta-Galactosidase (PA5-102503)	Thermo Fisher Scientific, Rockford, IL, USA	Primary antibody/Polyclonal	Rabbit/IgG	1:1000
Perlecan (MA1-06821)	Thermo Fisher Scientific, Kandel, Germany	Primary antibody	Rat/IgG2a	1:1000
Actin (MA1-744)	Thermo Fisher Scientific, Rockford, IL, USA	Primary antibody/Monoclonal	Mouse/IgG1	1:1000
Goat anti-rabbit IgG (H + L) HRP	Thermo Fisher Scientific, Rockford, IL, USA	Secondary antibody/Polyclonal	Goat/IgG	1:500
Goat anti-mouse IgG	Thermo Fisher Scientific, Rockford, IL, USA	Secondary antibody/Polyclonal	Goat/IgG	1:500

**Table 8:** Antibodies and protocols used for immunohistochemistry staining.

Antibody	Manufacturer	Clone	Monoclonal/ Polyclonal	Localization	Epitope Retrieval Condition	Host	Dilution
MMP13 (710311)	Thermo Fisher Scientific, Rockford, IL, USA	3HCLC	Polyclonal	cytoplasm	0.01 M sodium citrate buffer, pH 6.0 at 99–100 °C—30 min	Rabbit/IgG	pre-blocked with 3% BSA-PBS for 30 min at RT 1:100 overnight at 4 °C in a humidified chamber
Perlecan (MA1-06821)		A7L6	Monoclonal	extracellular matrix and basement membrane	0.01 M sodium citrate buffer, pH 6.0 at 99–100 °C—30 min	Rat/IgG2a	pre-blocked with 3% BSA-PBS for 30 min at RT 1:200 for 45 min at RT in a moisture chamber
Collagen II (PA1-26206)		-	Polyclonal	extracellular matrix structural protein	0.01 M sodium citrate buffer, pH 6.0 at 99–100 °C—30 min	Rabbit/IgG	pre-blocked with 3% BSA-PBS for 30 min at RT 1:400 for 45 min at RT in a moisture chamber
CD68 (MA5-13324)		KP1	Monoclonal	intracellular lysosomes of monocytes and macrophages and to a lesser extent by dendritic cells and peripheral blood granulocytes	0.01 M sodium citrate buffer, pH 6.0 at 99–100 °C—30 min	Mouse/IgG1, kappa	pre-blocked with 3% BSA-PBS for 30 min at RT 1:100 and incubated overnight at 4 °C in a moisture chamber
beta Galactosidase (PA5-102503)		-	Polyclonal	intracellular lysosomes enzyme	0.01 M sodium citrate buffer, pH 6.0 at 99–100 °C—30 min	Rabbit/IgG	pre-blocked with 3% BSA-PBS for 30 min at RT 1:50 for 1.5 h at RT in a moisture chamber

**Table 9:** Staining protocol for Ventana GX Benchmark.

Recommended Staining Protocol with UltraView Universal DAB Detection Kit (760–500) Ventana	
1.	Load slides, ultraView™ detection kit, and counterstain kit dispensers onto BenchMark^®^ instrument.
2.	Check the EZ Prep, Reaction Buffer, LCS, and Cell Conditioning Solution containers to be at least at 50% of their capacity.
3.	Start the run.
4.	When the staining run is complete, move the slides from the instrument and rinse well with a wash buffer.

**Table 10:** Troubleshooting Guide for IHC staining.

Problem	Possible Cause	Solution
Lack of tissue on slides at the end of IHC reaction	Problems with the processing protocol Incorrect use of slides Tissue cut too thick IHC protocol too aggressive with the tissue	The protocol must be very carefully designed to assure the tissue needs Positively charged or silane adhesive slides are required Some automated benchmarks can be incompatible with a new protocol
Spotty background	Aggregated secondary antibody Not enough washing between steps	Use fresh buffers Rince 3–5 times Improve the blocking step
High background	High concentration of the antibody Not enough blocking of endogenous peroxidase	Optimize primary and secondary antibody concentrations Improve the blocking protocol Use fresh buffers Increasing the washing time
Destroyed tissue morphology	Problems with the processing protocol Tissue cut too thick	Special attention to fixation and decalcification Hard tissues should be cut at 2 μm
Weak target staining	Low concentration of the antibody Degraded target or antibody	Increase the antibody concentration Test on a positive control

**Table 11:** Schematic protocol of the experiment.

Step	Description	Materials	Duration	Notes
Harvesting	Collect samples during knee arthroplasty	Surgical tools, sterile containers	2–8 h post-surgery	Ensure samples are from affected areas
Transport	Move samples to lab in sterile conditions	Sterile containers with PBS	Immediate	Prevent contamination and leakage
Maintenance	Wash and set up culture using sterile instruments	PBS, antibiotics, chondrogenic media or DMEM, serum free sterile Petri dishes	Up to 24 h post-collection	Maintain sterile conditions
Assessment	Western blot and IHC for protein analysis *	Western blot and IHC kits, specific antibodies **	Varies	Tailor protocols based on specific protein targets

* Availability of methods for high throughput quantitative assessment are to be envisaged for application in testing novel therapeutics, ** Antibodies adjusted to respective pathway of interest (e.g., inflammation, matrix degradation, matrix maintenance, chondrocyte viability, senescence).

## Data Availability

The data are available from the authors upon reasonable request. (Camelia-Mihaela Danceanu-Zara, cdanceanu@phys-iasi.ro).
